# New York State Veterinary Medical Association

**Published:** 1896-10

**Authors:** 


Secretary.

### NEW YORK STATE VETERINARY MEDICAL ASSOCIATION.

THE seventh annual meeting was held at the Genesee House, Buffalo, September 4 and 5, 1896.

### FIRST DAY, FRIDAY, SEPTEMBER 4TH, 11 A.M.

On roll-call there were sixteen members present, but within half an hour the parlors were well filled, about thirty-five members present, and fifteen to twenty visiting veterinarians from various parts of the United States dropped in to see and hear the proceedings. Among them were some of the lights in the profession.

The President opened the meeting with the annual address, which was listened to with unusual interest and well received. The Secretary's report was then read, which dealt entirely with the finances and business management of the Society. On motion, the report was accepted as offered and ordered spread on the minutes. The applications of twelve candidates were offered and referred to the Board of Censors.

A question was offered for discussion relative to present and future applicants when elected to the Society receiving their certificate of membership with the understanding that they should hold possession of the same during their continuing as members in good standing, and whenever that relation should cease that their certificate of membership be returned to the Society. Drs. George Berns, W. L. Baker, N. P. Hinkley, W. H. Kelly, John Wende, H. S. Wende, Wilson Huff, J. P. Thompson, and W. L. Williams, of the State Veterinary College at Cornell University, entered heartily into the discussion.

Dr. M. R. Trumbower, of Sterling, Ill., made a few remarks and invited the Society to attend their State meeting of veterinarians in November next, bringing with them any matter of interest to the profession at large. A vote of thanks was given Dr. Trumbower.

Dr. Osgood, of Massachusetts, made a few remarks on the status of the profession, which were well received.

Dr. Hoskins, of Philadelphia, spoke in his usual pleasing manner for a few minutes on the character of the profession in New York State.

Meeting adjourned for lunch.

AFTERNOON SESSION, FRIDAY, SEPTEMBER 4TH, 1.30 P.M.

The President called Dr. J. P. Thompson to the chair. Report of the Committee on Arrangements submitted, which included the arrangements for the United States Association as well as the State Society. The members were well pleased with what had been provided for their comfort and enjoyment, and wished to thank the committee for their efforts.

Committee on Legislation reported the following: "The past session of the Legislature has been a very interesting one for the veterinarians of this State, not so much on account of there being any such important bill passed as there was in the year of 1895, but that we have been able to hold this law in full force; and it has only been amended in behalf of the veterinarian who did not register according to law through some misunderstanding or oversight. There have been several attempts to set the law of 1895 aside temporarily in behalf of the non-graduate, but we are pleased that these efforts have been unsuccessful.

"During the session there were six bills introduced into the Legislature in which the veterinarians were interested: The first, Assembly Bill No. 249, was introduced January 16th by Hon. C. C. Cole, entitled An Act to amend the public health law, relating to the practice of veterinary medicine; the second, Senate Bill No. 227, was introduced January 21st by Hon. T. Sullivan, entitled An Act to amend sections number one thousand and eighty-one and eleven hundred and twenty-seven of the code of civil procedure; the third, Assembly Bill No. 346, introduced January 22d, the same as Senate Bill No. 227; the fourth, Senate Bill No. 351, introduced January 30th by the Hon. J. F. Ahern, the same as Senate Bill No. 227 and Assembly Bill No. 346; the fifth, Assembly Bill No. 995, introduced February 19th by Hon. James S. Harrison, entitled An Act to enable James Wixon of Steuben County to practise veterinary medicine and surgery as a profession; the sixth, Assembly Bill No. 2123, introduced April 1st by Hon. Uriah S. Messiter, entitled An Act to enable persons to register as practitioners of veterinary medicine and surgery in the State of New York, May 11, 1896, and who were qualified to register under said act and failed to do so; the seventh, Assembly Bill No. 2700, introduced April 21st by Hon. M. V. Ives, entitled An Act to amend Chapter No. 813 of the laws of 1896, entitled An Act to regulate the practice of veterinary medicine and surgery in the State of New York.

"The first, Assembly Bill No. 249, as originally introduced, would let any person register that was a graduate of a reputable college, or institution that claimed to be, as there were no restrictions. The committee seriously objected to this on that ground, and wrote several letters of protest and appeared in person before the Public Health Committee, stating their objections. After considerable debate the committee finally decided to report it favorably, as amended in Assembly Bill No. 1183. This met with the unanimous indorsement of the committee, and finally, after the Governor's signature had been affixed, became a law May 22, 1896.

"Senate Bill No. 227 was killed in the committee, as we were unable to get it reported. If it had become a law it would exempt the veterinarians of Kings and Queens Counties from jury-duty, which was an oversight in the passage of a similar bill last winter. Assembly Bill No. 346 was the same bill as Senate Bill No. 227. While it passed the Assembly it did not pass the Senate. Senate Bill No. 351 was the same as No. 227 and met the same fate.

"Assembly Bill No. 995 was introduced entirely in behalf of a quack named James Wixon, of Steuben County. While it was being acted upon in the Assembly the committee was not aware of it. Attention was called to it by Dr. A. O'Shea. Upon investigation, it was found that the bill had passed the Assembly and reached the Senate and was in the hands of the committee. Immediately several members throughout the State were notified, and Drs. Morris and O'Shea, on the receipt of theirs, each had some two hundred circular letters printed, stating the nefarious condition of the bill, and also wrote the Chairman of the Committee.

"Assembly Bill No. 2123 was entirely in behalf of the non-graduates, and was another one of their attempts to set aside the law. Assembly Bill No. 2709 was also another struggle of the dying quacks, which was also killed."

A vote of thanks was given to the committee for their effective and excellent work.

(To be continued.)

